# School attendance among refugee children with disabilities residing in South Africa: A cross-sectional, descriptive study

**DOI:** 10.1371/journal.pone.0279671

**Published:** 2023-05-19

**Authors:** Nicole De Wet-Billings, Khuthala Mabetha

**Affiliations:** Faculty of Humanities, Demography and Population Studies, School of Social Sciences, University of the Witwatersrand, Johannesburg, South Africa; Jashore University of Science and Technology (JUST), BANGLADESH

## Abstract

Refugee children with disabilities are entitled to an education under South African law. These children face the challenges of living in a different country and having to manage their disabilities. However, without providing a quality education to refugee children with disabilities, they face lifelong challenges including poverty and exploitation. This nationally representative cross-sectional study, examines the prevalence of school attendance of refugee children with disabilities in South Africa. Using the Community Survey of 2016, 5,205 refugee children with disabilities are identified and studied. Descriptive statistics are used and results show that less than 5% of refugee children with disabilities are in school. Further there are differences across province of residence, sex and other sociodemographic characteristics. This study is a starting point for more quantitative analysis and further qualitative analysis on the barriers to education for refugee children with disabilities in the country.

## Introduction

Access to education is a human right. However, globally, as many as 8% of children of primary school age are not in school [1]. In sub-Saharan Africa, 19% of children do not attend school and by the time they reach adolescence, girls have a 36% exclusion rate compared to 32% for boys [1,2]. Children are denied access for a number of reasons including cultural and gendered practices, socioeconomic inequalities, lack of funding for and investment in schools, poor infrastructure, lack of staff and learning materials and due to learning and other disabilities [2–5].

For children with disabilities, the challenges at mainstream schools are many. First, the infrastructure does not cater for the needs of children with disability, including not having ramps and elevators for children with walking disabilities, no assistance around the property or alternative learning means for visually impaired children and so forth [5–7]. The cost to implement these upgrades at public schools is often too high for governments and local municipalities to bear. Second, mainstream schools do not have teachers and staff trained to assist children with various types and degrees of disability [8,9]. The teachers who are specially trained for children with special needs, including disability, are not many. In South Africa, only 43% of teachers have been trained on setting assessments for learners with a learning disability [10]. Third, children with disabilities are viewed as a burden on the education system. A study on visually impaired school children in Ghana found that teachers reported pupils with disability slowed down the lessons and impeded progress for non-disabled pupils in class [11].

There does exist, parallel to the mainstream school system, schools which are designed and staffed to assist children with disabilities. However, in many instances, including in South Africa, these schools are at an additional cost for parents and caretakers [12–14]. The country has many schools for children with disability and these are exempt from the no-fee policy of education in the country, which means that fees are not wholly subsidised by the government [14]. In addition to the cost of these schools, parents struggle to afford transport, and are subject to waiting lists for enrolment [15,16].

There also exists social factors outside of the school system that prohibit children with disabilities from accessing education. One factor is migration status and for many children, with and without disabilities, language and cultural differences contribute to non-attendance [17]. Xenophobia is another major barrier with one study on Zimbabwean migrant children in South African schools finding demonstrable evidence of discrimination based on country of birth [18]. And related to migration status are the rules and regulations around refugees. Almost every region of the world differs in terms of its treatment of refugees, with some countries providing housing, employment and education options while others simply keep refugees in camps until laborious paperwork is completed which could take years to do. In South Africa, refugees are not housed in camps, but social and economic assistance is not favourable either [19]. According to government policy in South Africa, refugees are entitled to seek employment and the same basic health services and basic primary education which the inhabitants of the Republic receive [20]. However, the reality for many refugees does not reflect this, with xenophobic attitudes preventing many refugees from accessing employment, health care and education [20].

For refugee children with disabilities, the extent to which exclusion from education remains largely unknown. The purpose of this study is to identify the prevalence of refugee children with disabilities in South Africa and to profile the school attendance given a number of sociodemographic characteristics of the refugee children.

## Data and methods

### Data source

The study uses data from the South African Community Survey 2016. This is a large-scale survey that happened between Censuses 2011 and 2021. The main objective of the survey is to provide population and household statistics at municipal level to government and the private sector, to support planning and decision-making between Census periods and approximately 1.3 million households were sampled in this survey (https://www.statssa.gov.za/?page_id=6283).

### Study population and sample

The study population are children aged between 7 and 18 years old whose status in the country at the time of the survey was as refugees. That is, not born in South Africa and have immigrated to the country due to violence, unrest or political reasons. The total study population is 106 638 (weighted) refugee children aged 7–18 years old. Of this population, this study is particularly interested in the refugee children with at least one type of disability. The survey asks respondents, who are parents and caretakers for these minor- aged children, if the child in their care has any of six listed disabilities. The sample of refugee children with a reported disability in this study is 5,205 (weighted). These children are included in the study.

### Study variables

The outcome of interest to this study is the disability status of refugee children. In this study, we quantify each type of the six listed disability options which are sight, hearing, communication, remembering, walking and self- care. Also a binary variable is created which captures if each child has at least one type of disability (yes or no).

The independent variables in this study are the sociodemographic characteristics of the refugee children. These are region of birth, age-group (7–10, 11–14 and 15–18 years old), sex (male or female), province of residence, educational institution and type of institution (public or private). The latter two variables relate to the level of education and cost of education for the parents and caregivers of refugee children with disabilities. Also included is the main independent variable ‘school attendance’ which is derived from the survey question ‘Does (the child) presently attend an educational institution?’ with positive responses are coded as ‘yes’ and negative responses are coded as ‘no’.

### Analysis

Due to the study sample of refugee children with disability being low at less than 5% of all the refugee children in the country, only descriptive statistics have been produced using the data. Cross-tabulations and chi-square analysis have been used to determine the prevalence of disability among refugee children by various sociodemographic characteristics, including school attendance.

## Results

**Fig 1:**
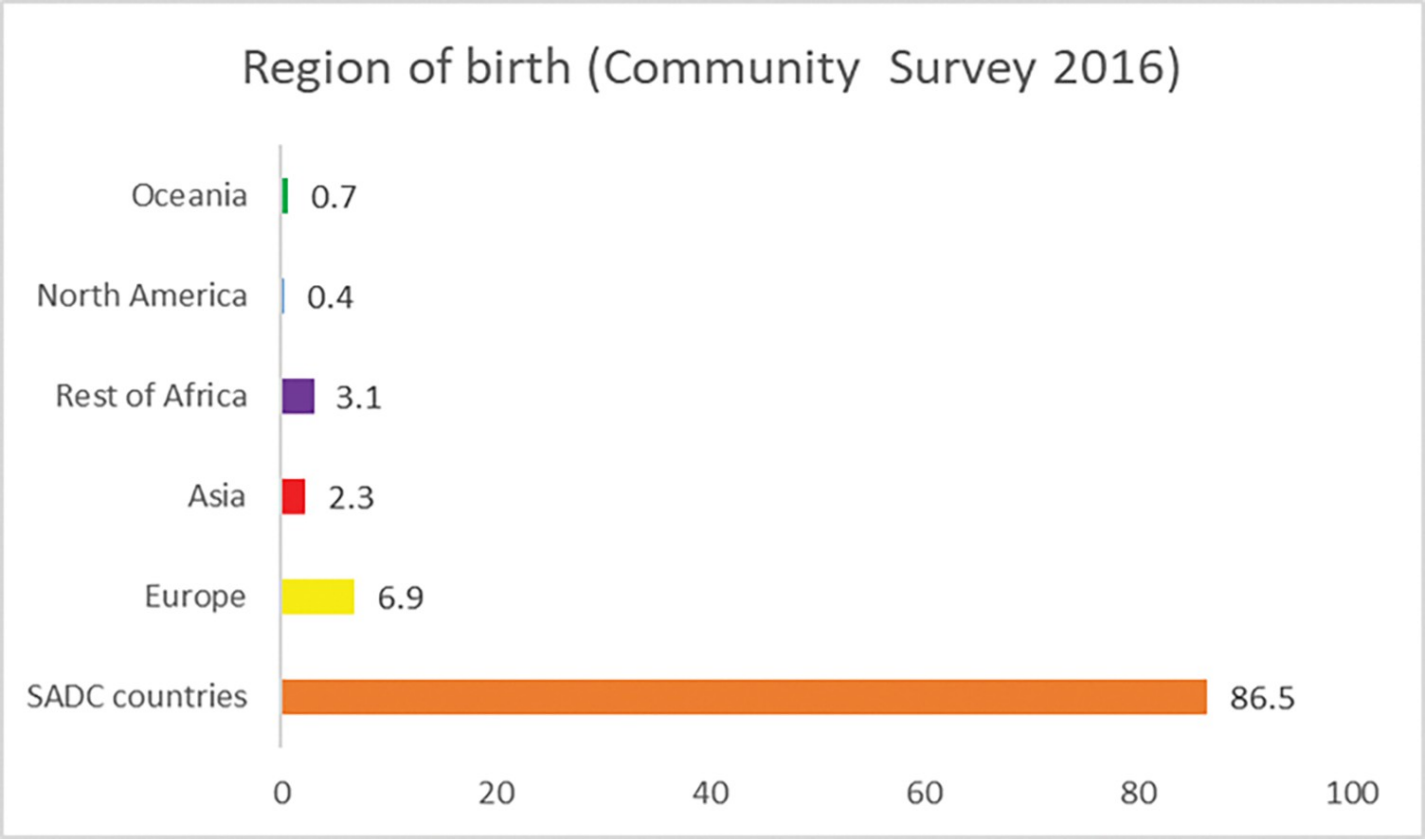
Region of birth of refugee children (7–18 years old) in South Africa, 2016.

Similarly, majority (86%) of refugee children were reported to have originated from countries in the SADC region (Fig 1), followed by 7% who were reported to have originated from Europe. The lowest percentage was observed among refugee children who were reported to have originated from North America.

**Fig 2:**
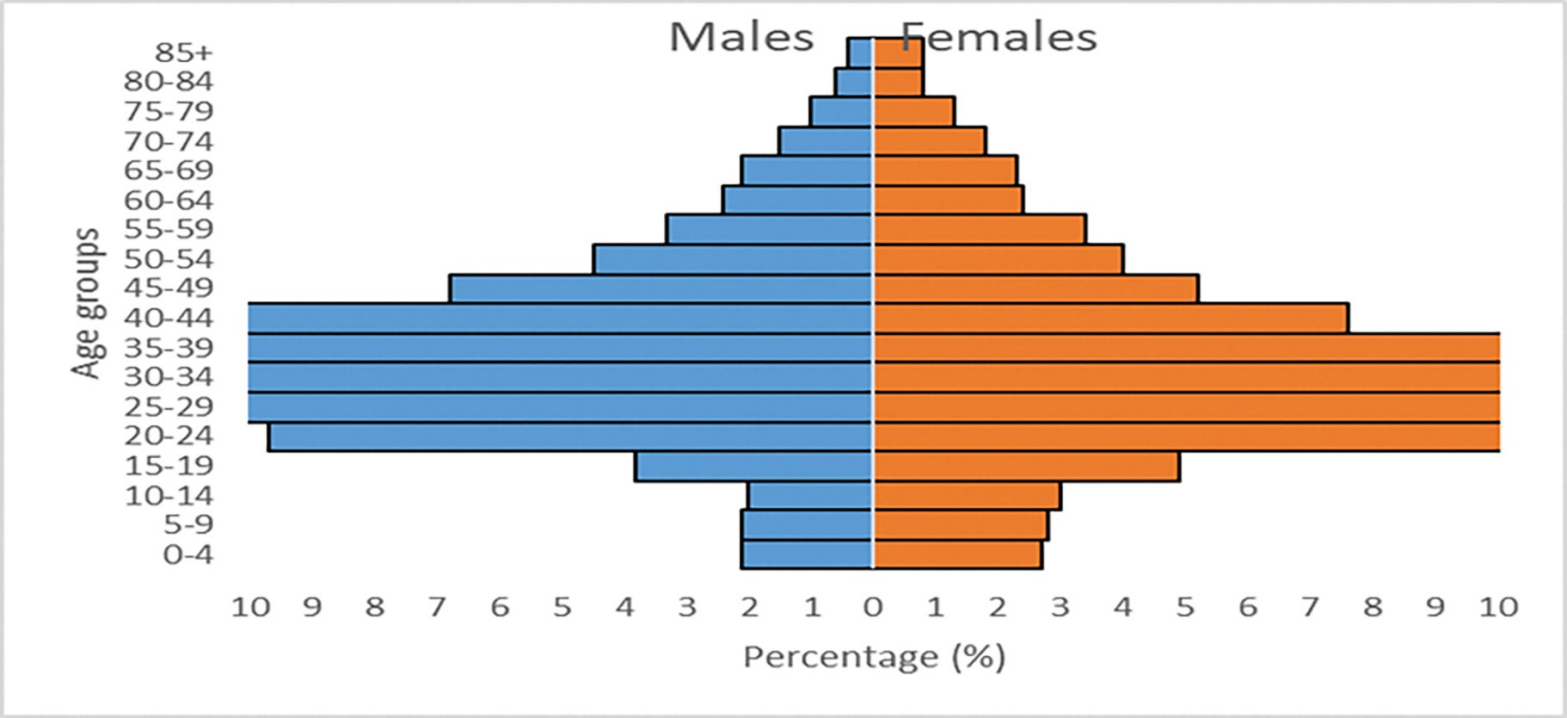
Age-sex composition of refugee population residing in South Africa, 2016.

Fig 2 shows the age and sex composition of the refugee population residing in South Africa. Among the younger age groups, the figure shows that about 8% are male and approximately 10% are female. This means that there is an early age dependency of almost 20% of the refugee population being under the age of 15 years old.

**Fig 3:**
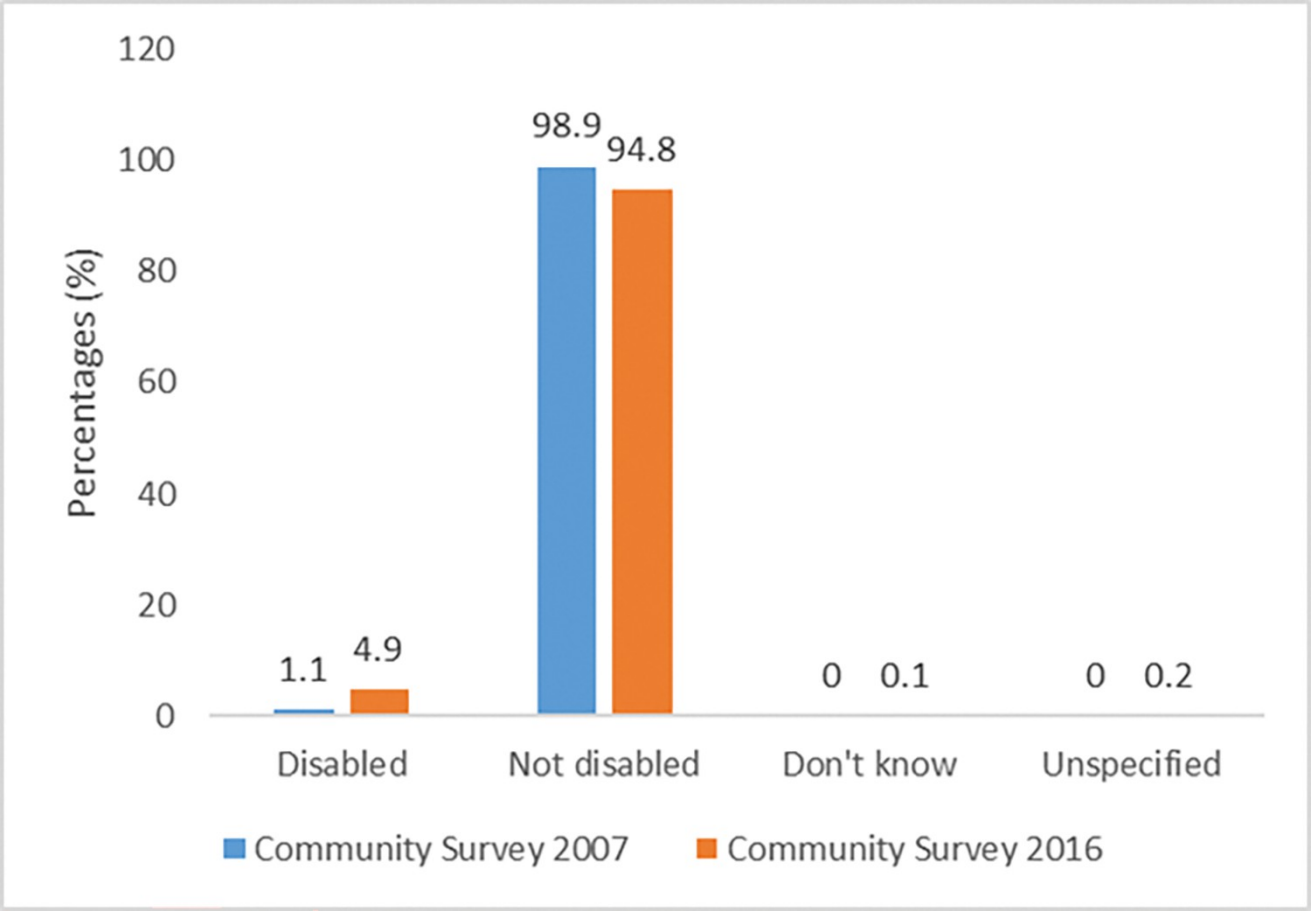
Disability status of refugee children (7–18 years old) in South Africa, Community Survey.

Fig 3 shows the disability status of refugee children of school-going age in South in 2007 and 2016. The results show that in 2007, only 1.1% of the sampled population of refugee children were living with a disability. This increased to almost 5% of refugee children who were reported to have a disability in 2016.

**Fig 4:**
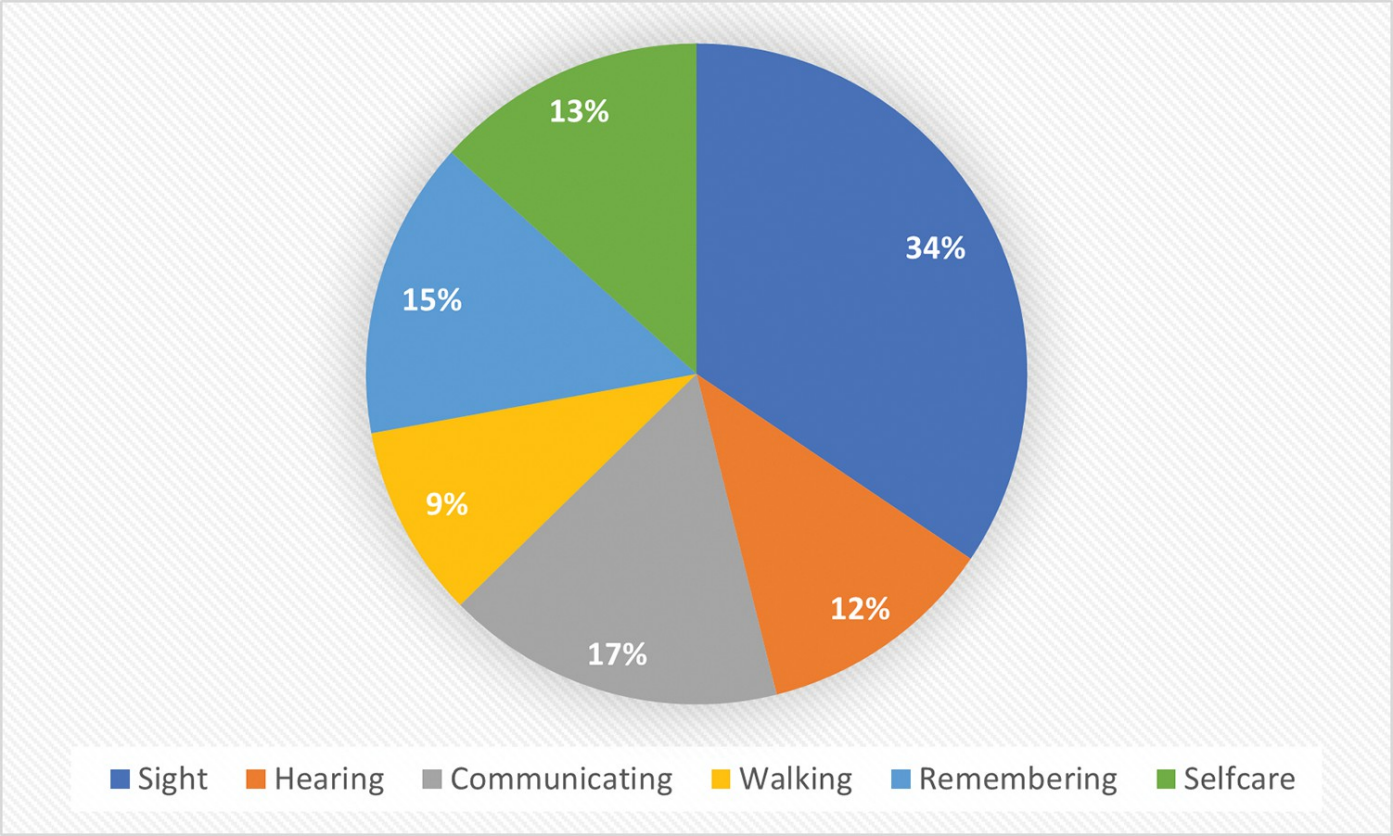
Percentage distribution of type of disability reported (N = 5,205) among refugee children (7–18 years old) residing in South Africa, 2016.

Among the reported disabilities of children in the survey, 34% have a sight disability (Fig 4), 17% have at least some difficulty communicating and 15% have a remembering disability. Further, 13% have at least some difficulty with self-care, 12% have a hearing disability and 9% have trouble walking.

A total of 21.54% of refugee children with disability are not attending school and 78.27% are attending school (Table 1). Of the children with a sight disability, 17.43% are not attending school. Further, 31.89% of children with hearing, 46.92% with communicating, 48.38% with walking, 31.84% with remembering and 33.49% with self-care disabilities are not attending school.

**Table 1:** Frequency and percentage distribution of type of disability by school attendance among refugee children (7–18 years old) residing in South Africa, 2016.

Type of Disability	School Attendance						
	Yes		No		Do not know		Total
	Frequency (N)	Percentage (%)	Frequency (N)	Percentage (%)	Frequency (N)	Percentage (%)	Frequency (N)
**TOTAL**	**83 470**	**78.27**	**22 968**	**21.54**	**201**	**0.19**	**106 638**
**Sight** *							
No	80 671	78.19	22 343	21.66	155	0.15	103 170
Yes	2 695	82.57	569	17.43	0	0.00	3 263
Unknown	104	50.74	56	27.31	45	21.96	205
**Hearing** *							
No	82 613	78.43	22 560	21.42	155	0.15	105 328
Yes	752	68.11	352	31.89	0	0.00	1 105
Unknown	104	50.74	56	27.31	45	21.96	205
**Communicating** *							
No	82 532	78.70	22 176	21.15	155	0.15	104 863
Yes	833	53.08	736	46.92	0	0.00	1 569
Unknown	104	50.74	56	27.31	45	21.96	205
**Walking** *							
No	82 867	78.56	22 465	21.30	155	0.15	105 487
Yes	462	51.62	433	48.38	0	0.00	895
Unknown	141	54.95	70	27.44	45	17.61	256
**Remembering** *							
No	82 411	78.47	22 460	21.38	155	0.15	105 026
Yes	941	68.16	440	31.84	0	0.00	1 381
Unknown	118	50.91	69	29.62	45	19.46	232
**Self- care** *							
No	82 502	78.46	22 490	21.39	155	0.15	105 148
Yes	838	66.51	422	33.49	0	0.00	1 260
Unknown	130	56.16	56	24.30	45	19.54	231

*denotes p-value <0.05.

Of the refugee children living in South Africa, 4.88% have a disability (Table 2). Among the female children, 5.54% and among the male children, 4.19% have a disability. By age-group, 4.33% of 7 to 10 year olds, 5.59% of 11–14 year olds and 4.76% of 15–18 year old refugee children have at least one disability. The Eastern Cape province (8.88%) reported the highest percentage of refugee children with disability, while the Western Cape province (3.22%) reported the lowest percentage across the country’s regions. Table 2 further shows that less than 5% of the refugee children with at least one disability are in school and 8.24% are at home-based institutions of education. Finally, 5.03% of refugee children with disability are at public institutions of learning and 4.47% attend private institutions.

**Table 2:** Sociodemographic profile of refugee children (7–18 years old) residing in South Africa by disability status, 2016.

Characteristics	Total	Disability status—Yes		Disability Status—No	
	Frequency (N)	Frequency (N)	Percentage (%)	Frequency (N)	Percentage (%)
**Total**	**106 638**	**5 205**	**4.88**	**101 433**	**95.12**
**Sex** *	*** ***				
Female	54 751	3 033	5.54	51 718	94.46
Male	51 887	2 172	4.19	49 714	95.81
**Current age** *	*** ***				
7–10 years	32 005	1 386	4.33	30 619	95.67
11–14 years	32 033	1 792	5.59	30 241	94.41
15–18 years	42 600	2 027	4.76	40 573	95.24
**Province** *	*** ***				
Western Cape	16 628	536	3.22	16 092	96.78
Eastern Cape	4 774	424	8.88	4 350	91.11
Northern Cape	846	59	6.98	786	93.01
Free State	3 615	263	7.28	3 352	92.73
KwaZulu-Natal	6 091	341	5.60	5 750	94.40
North West	7 037	397	5.64	6 640	94.36
Gauteng	48 548	2 408	4.96	46 140	95.04
Mpumalanga	9 608	381	3.97	9 227	96.04
Limpopo	9 491	396	4.17	9 094	95.82
**School attendance** *					
Yes	83 470	4 078	4.89	79 392	95.11
No	22 968	1 128	4.91	21 840	95.09
Don’t know	201	0	0	201	100.00
**Educational Institution** *		*** ***			
Pre/Primary	50 538	2 398	4.75	48 140	95.25
Secondary	31 494	1 622	5.15	29 873	94.85
Tertiary/college	717	13	1.87	703	98.13
Home-based	367	30	8.24	337	91.76
Other	23 522	1 142	4.85	22 380	95.15
**Public or Independent institution** *					
Public	61 788	3 108	5.03	58 680	94.97
Private	21 161	947	4.47	20 214	95.53
Other	23 689	1 150	4.86	22 538	95.14

*p-value<0.05.

**Fig 5:**
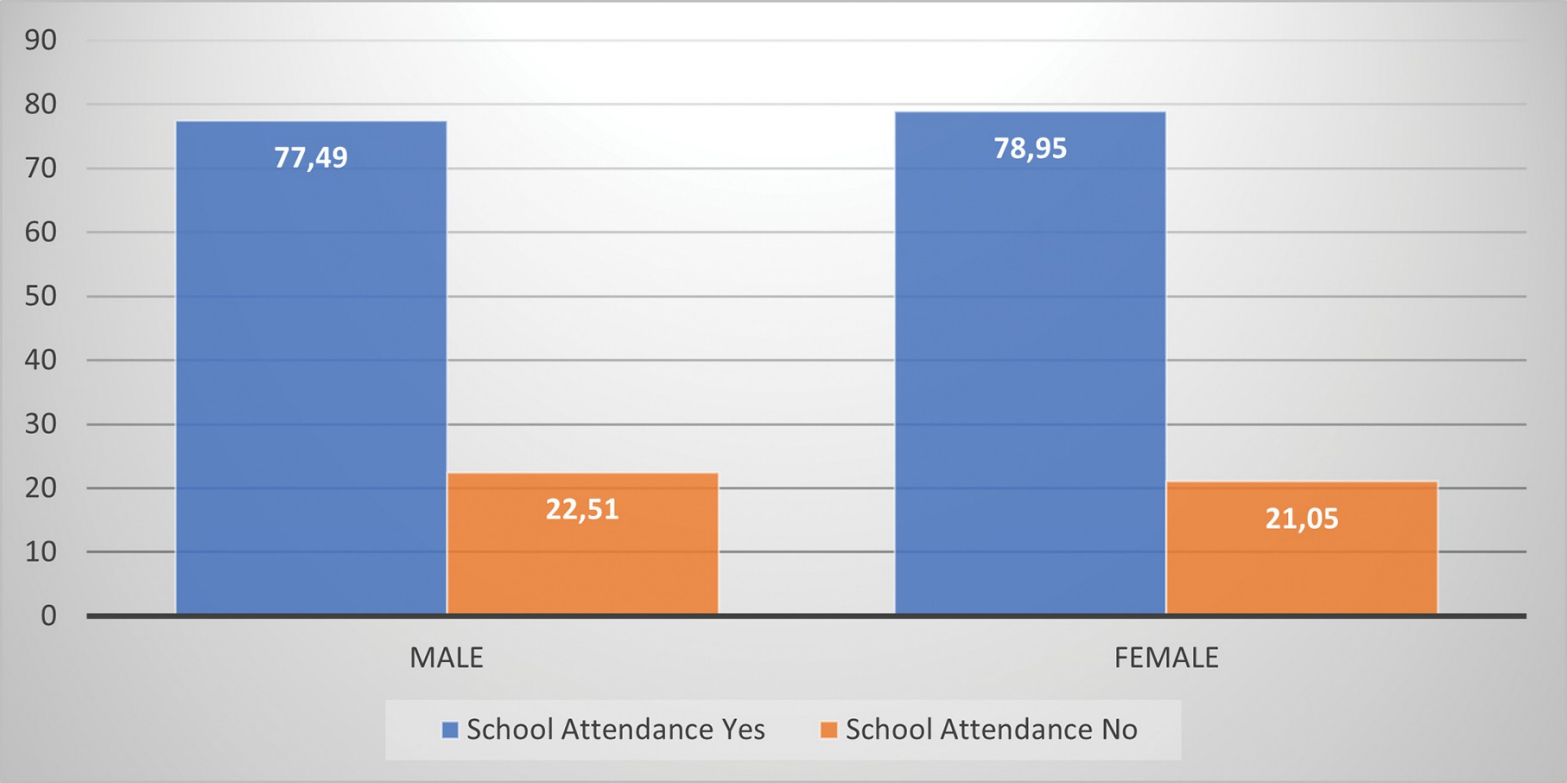
Percentage distribution of refugee children with disability residing in South Africa by school attendance status and sex, 2016.

Fig 5 shows that 77.49% of male refugee children with at least one disability residing in South Africa attend school and 78.95% of their female counterparts are also in school. This leaves 22.51% of male and 21.05% of female refugee children with disabilities not attending schools in South Africa.

## Discussion

The purpose of this study is to highlight the prevalence of refugee children with disabilities’ school attendance in South Africa. With every child having the right to an inclusive education experience, there is no reason why any should not be in school. The study however has found that almost 5% of refugee children with disabilities do not attend school. Globally, it is estimated that 61% of refugee children do not attend school and of these 15% to 20% have disabilities [21,22]. For many of these children, education or schooling is offered in refugee camps, which have their own challenges including the lack of personnel, resources and infrastructure in these settlements, but in South Africa, where there are no camps, refugees are meant to join mainstream schools for education [22,23]. Schools, in general, are meant to be inclusive environments that encourage learning, teach pupils how to interact and allow young people to grow. For refugee children, schools are meant to facilitate learning and transitions for refugee children to citizenship and enable them the opportunity to acquire skills for economic purposes. However, schools in South Africa are plagued with issues of xenophobia, under-staffing and inadequate resources. This would result in many refugee parents keeping their children out of school.

By type of disability, more than 15% of refugee children with at least one type of disability are not attending school. This is related to the problems of inclusive education that exist in South Africa. One study has revealed that funding problems, lack of commitment to brace inclusion and inclusive policies, and training of teachers in inclusive education are major barriers to combat xenophobia and assist children with disabilities in attending mainstream schools [24]. Since this study was done, many others in the country have revealed similar challenges and very little progress [17,25–27].

Older refugee children with disabilities are not in school more than younger children. A recent UNHRC report found a similar result with secondary school attendance for refugee children being lower than primary education [21]. Possible reasons why older refugee children might not be attending school could be similar to those of non-refugee children which include financial constraints on the household, the need for older children to seek employment to assist families and drop-out due to non-progression or failing grades [28–31]. This compromises young people’s ability to obtain skills and knowledge that would, in the long run, enable these children to obtain gainful employment.

There is also a gendered discrepancy in refugee children with disabilities not attending school. With fewer female children than males in school. Gendered differences in school attendance is not surprising in African countries, and others, where male children are given preference for education over girl children [32–35]. However, the vulnerability of female refugee children with disabilities not attending school cannot be understated. These young girls will be limited in employment opportunities and face the hardship of poverty, exploitation and further health challenges if they are unable to attend school and obtain an education.

The study is subject to a few limitations. First, the refugee status of participants is not directly asked and rather derived from questions pertaining to place of birth and reason for moving to South Africa. A more direct question might have wielded a bigger sample of refugees. Second, the reporting of refugee children with disabilities might be an under-count given that (1) many refugees and foreign nationals in South Africa fear xenophobic violence (REF) and would not disclose their migrant status and (2) this would lead to children with disabilities being counted as citizens and not refugees.

There are also a few strengths to the study. The data are nationally representative and therefore a larger sample of refugee children with disabilities are captured than what would have been possible otherwise. The study is also able to produce reliable (p-values <0.05) descriptive statistics on the school attendance and sociodemographic profile of refugee children with disabilities. Finally, the study is a starting point for statistical and qualitative analysis on the barriers to inclusive education for refugee children with disability in South Africa. The country is unique in the region because of the lack of refugee camps and the struggling education system. Future studies on this could assist in strengthening inclusion policies in South Africa in both the social and education systems.

In conclusion, refugee children with disabilities are not all attending school. But they should be. These children’s needs should be accommodated in mainstream schools, if their disabilities are minor, and in special schools for more severe cases. However, cost and xenophobia need to be addressed in the education system in order to achieve inclusive education across the country for refugees who reside in various parts of the country.
